# Inter-Dye Distance Distributions Studied by a Combination of Single-Molecule FRET-Filtered Lifetime Measurements and a Weighted Accessible Volume (wAV) Algorithm

**DOI:** 10.3390/molecules191219269

**Published:** 2014-11-25

**Authors:** Henning Höfig, Matteo Gabba, Simón Poblete, Daryan Kempe, Jörg Fitter

**Affiliations:** 1 I. Physikalisches Institut (IA), RWTH Aachen University, Otto-Blumenthal-Straße, 52074 Aachen, Germany; E-Mails: hoefig@physik.rwth-aachen.de (H.H.); kempe@physik.rwth-aachen.de (D.K.); 2 Institute of Complex Systems (ICS-5), Molecular Biophysics, Forschungszentrum Jülich, Wilhelm-Johnen-Straße, 52428 Jülich, Germany; 3 Institute of Complex Systems (ICS-2), Theoretical Soft Matter and Biophysics, Forschungszentrum Jülich, Wilhelm-Johnen-Straße, 52428 Jülich, Germany; E-Mail: s.poblete@fz-juelich.de

**Keywords:** smFRET, lifetime FRET measurements, dye-linker dynamics, protein dynamics

## Abstract

Förster resonance energy transfer (FRET) is an important tool for studying the structural and dynamical properties of biomolecules. The fact that both the internal dynamics of the biomolecule and the movements of the biomolecule-attached dyes can occur on similar timescales of nanoseconds is an inherent problem in FRET studies. By performing single-molecule FRET-filtered lifetime measurements, we are able to characterize the amplitude of the motions of fluorescent probes attached to double-stranded DNA standards by means of flexible linkers. With respect to previously proposed experimental approaches, we improved the precision and the accuracy of the inter-dye distance distribution parameters by filtering out the donor-only population with pulsed interleaved excitation. A coarse-grained model is employed to reproduce the experimentally determined inter-dye distance distributions. This approach can easily be extended to intrinsically flexible proteins allowing, under certain conditions, to decouple the macromolecule amplitude of motions from the contribution of the dye linkers.

## 1. Introduction

The fluorescence lifetime is perhaps the most sensitive parameter in fluorescence spectroscopy [1]. Owing to the lifetime, organic fluorophores are sensitive reporters about the surrounding environment and the solvent properties. Kinetic processes affecting this quantity on a timescale slower than a few nanoseconds are easily detected by virtue of the lifetime. Nevertheless, one of the most intriguing characteristics of the lifetime is its sensitivity to inter-dye distance changes, when two fluorophores, a donor and an acceptor, are coupled by Förster resonance energy transfer (FRET) [2]. In fact, this allows one to recover inter-dye distance distributions with Angstrom spatial-resolution and nanosecond temporal resolution. This was shown in the past by time-resolved ensemble FRET measurements [3,4] aiming to characterize the inter-domain motions of proteins [5,6,7], the conformational flexibility of oligopeptides [8] and, more recently, dye-linker motions [9]. In this respect, ensemble measurements of the donor lifetime in the presence of the acceptor are even better than single-molecule FRET experiments. For instance, motions faster than a few tens of microseconds are completely averaged out and cannot be detected from the efficiency histograms [10]. Indeed, only mean values of the inter-dye distance distributions are recovered. However, a general drawback of ensemble lifetime FRET measurements is the presence of unknown fractions of donor-only molecules, *i.e.*, of molecules missing an active acceptor. Indeed, donor-only components may bias the parameters describing the inter-dye distance distribution and hide low energy transfer populations, because the distribution width and mean values are strongly correlated with the donor-only amplitude [1]. Therefore, the removal of the donor-only population or an independent quantification of its contribution is strongly recommended in order to improve the estimation of the inter-dye distance distributions and to exploit the full power of lifetime FRET measurements.

**Figure 1:**
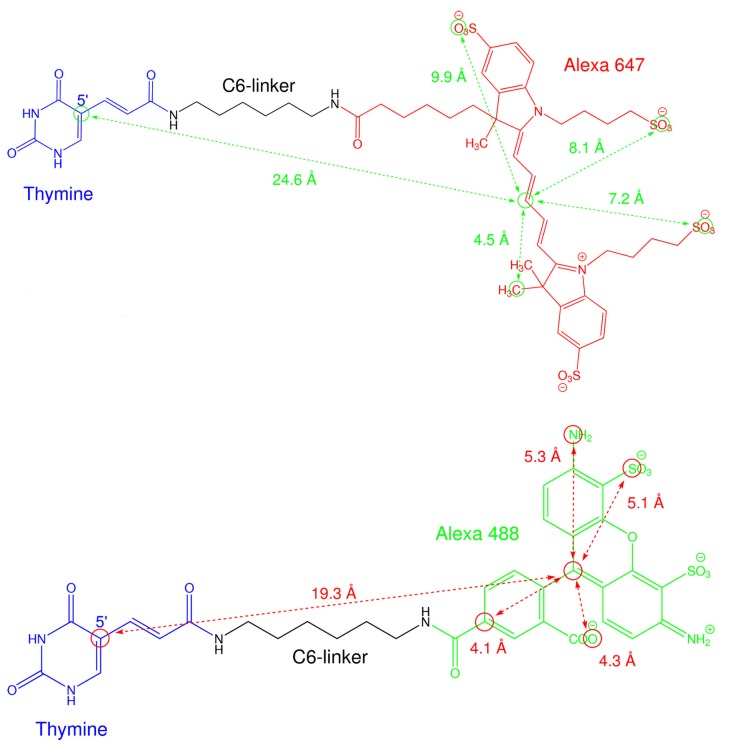
Chemical structure of two fluorophores used as a FRET pair (Alexa 647 in red and Alexa 488 in green) attached to DNA at position 5' of a thymine (T in blue) via a C6 amino linker (NH_2_-C_6_H_12_-NH_2_ in black). The distances (given by the arrows) are employed in Section 3.8 for coarse graining the dyes.

A second difficulty shared by single-molecule and ensemble FRET measurements is the presence of long flexible linkers (with a length of *∼*20 Å), connecting the fluorophores with the biomolecule (see Figure 1). These linkers are aliphatic chains inserted as spacers between the dye and the biomolecule in order to avoid sterical clashes that may hinder the labeling reaction and the free rotation of the dyes. Thereby, the presence of flexible linkers has remarkable effects on the measured distance distributions, as recently shown for intrinsically-rigid model systems, like double-stranded DNA molecules [9] and polyproline chains [11]. It was shown in these studies that the average dye positions are effectively shifted with respect to the label attachment points, biasing the mean inter-dye distances. In addition, the fluorophores diffuse randomly on fast timescales (*∼*100 ns) [12] within the sterically accessible volume, resulting in a large contribution to the width of the inter-dye distance distributions. Therefore, a methodical procedure is required to extract the contribution of the dye-linker dynamics from the experimental data. Different approaches were proposed, based on a conformational statistics description of the dye-linker complex [7] and simulations [11,13,14]. However, the recently proposed accessible volume algorithm [15,16] is probably the best compromise between simplicity and accuracy. This approach was originally developed for FRET-restrained structural modeling of biomolecules [15] and delivers all of the sterically accessible points given the spatial extension of the dye and the biomolecule structure. In this respect, the algorithm was optimized for calculating the mean inter-dye distances. Thus, it is not surprising that the amplitudes of motion predicted by this approach are overestimated as compared to those obtained from MD simulations [9]. Therefore, a different algorithm has to be envisioned in order to improve the calculation of the dye-linker contribution to the total amplitude of motions obtained from lifetime FRET measurements. Most importantly, this algorithm can finally be employed to unravel the dynamics of biomolecules, for example in the case of the inter-domain motions of enzymes [5,17].

In the present work, we performed a methodological study on double-stranded DNA (dsDNA) labeled with a FRET pair. This choice allows us to experimentally characterize the dye-linker dynamics in the absence of any other motions [9]. Indeed, since the persistence length of DNA (*∼*500 Å) [18] is one order of magnitude larger than the Förster radius (*∼*50 Å), dsDNA effectively behaves as a rigid rod on the characteristic length scales sampled by FRET. The use of pulsed interleaved excitation [19] to sort out donor-only molecules allows us to build up lifetime histograms without donor-only photons. Consequently, the precision of the inter-dye distance distribution improves with respect to common ensemble lifetime measurements. For instance, with the filtered decays, we were able to detect the effects of a base pair mismatch that was introduced in proximity of the labeling position of one of the dsDNA samples.   Furthermore, we modify the accessible volume (AV) algorithm [9] by usinga semi-empirical Gaussian probability distribution of the dye to calculate a more realistic accessible volume. This distribution reduces the impact of fully-extended conformations of the linkers due to hydrophobicity [20] and to conformational entropy [9] of the aliphatic chains. In this respect, we describe the dye-linker as an ideal chain with an effective bond length, which is experimentally calibrated with our DNA standards. By employing the same fluorophores in calibration and in application studies, this approach allows one to decouple the dye linker dynamics from internal protein motions. This was recently demonstrated for phosphoglycerate kinase [17], where the inter-domain amplitude of motion was recovered by means of the proposed weighted accessible volume (wAV) algorithm.

## 2. Results and Discussion

### 2.1. Ensemble Lifetime FRET Measurements

**Figure 2:**
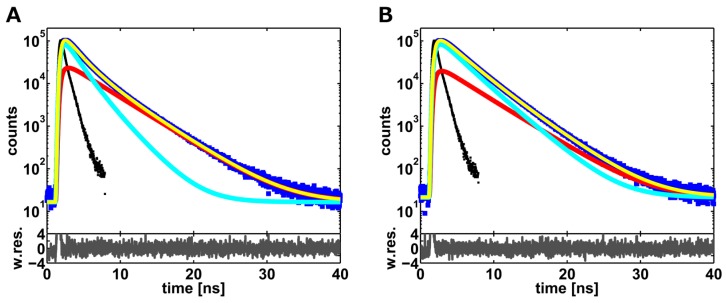
Donor ensemble lifetime decays and fitted model functions for: (**A**) 10 bp DNA and (**B**) 17 bp DNA. The model function, the sum of the donor-only component (red line) and the FRET-quenched decay (cyan line), is shown in yellow. The instrument response function (IRF) is presented in black (FWHM *∼* 544 ps.) The weighted residuals between the experimental data (blue squares) and the model function are shown in gray. For more details about the fits, see Section 3.5. The fitting parameters are reported in Table 1.

In this subsection, the pitfalls and limitations of ensemble lifetime FRET measurements are discussed based on two examples and by employing the analysis of artificial lifetime decays. First, to exclude possible polarization artifacts on the lifetime decays, which we recorded with one detector, measurements with D-only DNA samples were performed.  In Fisz *et al.* [21], it is shown that for a detection cone approaching 70*°*, the unpolarized lifetime decay equals *I*_⊥_ + *I_‖_*. In our set-up, a value of 64*°* is used, which is pretty close to the ideal one. Thus, we compared the lifetime decay measured with one detector to that one obtained by adding up the parallel (*I_‖_*) and perpendicular (*I*_⊥_) components recorded with two detectors and a polarizer cube. The relative deviation between the fitting parameters obtained in both cases is below *∼*2%, showing that the use of a “magic angle” set-up can be omitted. This is of particular importance in single-molecule experiments (see the next subsection), since a loss of almost 40% of photons introduced by the polarizing optical elements cannot be tolerated. Second, to rule out sticking of the fluorophores to the DNA grooves, as reported earlier by other groups [22,23], the anisotropy decays were measured for single-labeled DNA samples. In addition to a slow component visible in a five- to seven-nanosecond time regime, due to DNA tumbling, a faster decay in a one-nanosecond time regime (*∼*1.1 ns for Alexa 647 and *∼*0.9 ns for Alexa 488) was observed, indicating free rotation of the dyes. Thus, sticking of the fluorophores to DNA can be excluded. Based on these conditions, ensemble lifetime FRET experiments were performed and analyzed. In Figure 2, donor lifetime decays measured in ensemble for two DNA samples with labeling positions separated by 10 and 17 base pairs (bp) are reported. Both lifetime decays were fitted with the model function described in Equation (9) (yellow line), which considers a donor-only fraction *x_D_*_0_ (red line) and a Gaussian distribution of inter-dye distances (cyan line). This is a good approximation of the real distribution, as shown in [9]. The resulting fitting parameters are given in Table 1. For both samples, we obtain a fraction of donor-only molecules of *∼*17%. As expected, the inter-dye distance distribution of the 17-bp DNA sample is peaked at a higher mean inter-dye distance of (60.0 *±* 0.3) Å, as compared to the 10-bp DNA sample, where a distance of (46.2 *±* 0.2) Å is measured. In contrast, the obtained widths of the distributions are pretty similar, with values of (5.9 *±* 0.5) Å for 17-bp DNA and of (7.1 *±* 0.3) Å for 10-bp DNA samples.

**Table 1:** Fitting results obtained from ensemble and smFRET-filtered lifetime measurements performed with 10-bp and 17-bp DNA samples.

Sample	Measurement	〈*R_DA_*〉 [Å]	*σ_DA_* [Å]	*x_D_*_0_ [%]	$\chi_{r}^{2}$
10 bp DNA	ensemble	46.2 ± 0.2	7.1 ± 0.3	16.0 ± 0.3	1.033
10 bp DNA	smFRET filtered	49.1 ± 0.2	4.5 ± 0.4	4.8 ± 0.4	1.123
17 bp DNA	ensemble	60.0 ± 0.3	5.9 ± 0.5	18.2 ± 1.1	0.999
17 bp DNA	smFRET filtered	62.7 ± 0.2	7.2 ± 0.5	0	1.075

**Figure 3:**
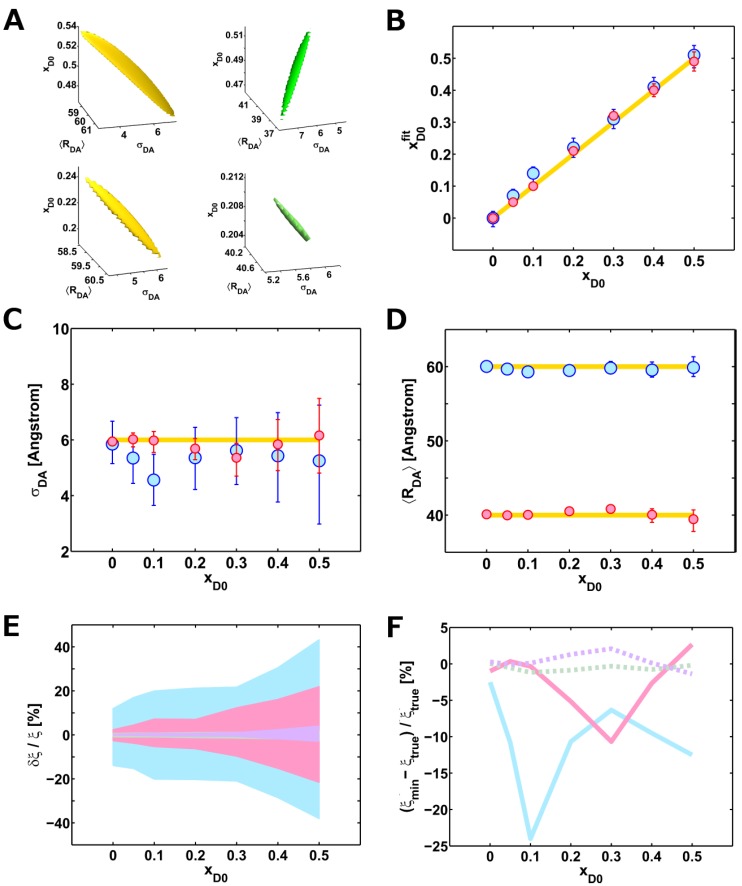
Results from the analysis of the artificial lifetime decays. (**A**) Representative set of confidence surfaces at one standard deviation (68.3%) from the minimum calculated for low (yellow) and high (green) FRET efficiencies. The donor-only fractions *x_D_*_0_ were 0.5 (**top**) and 0.2 (**bottom**). (**B**–**D**) Estimated parameters (*x_D_*_0_, *σ_DA_* and 〈*R_DA_* 〉) plotted as a function of the donor-only fraction for high (pink) and low (light blue) FRET efficiencies. The yellow lines represent the true values. The error bars are also indicated. The percentage relative errors (**E**) and accuracies (**F**) are shown as a function of the donor-only fraction for different true parameter values and FRET efficiencies. Color code: (i) *ξ* = *σ_DA_*; low FRET state (light blue) and high FRET state (pink); and (ii) *ξ* = 〈*R_DA_*〉; low FRET state (light green) and high FRET state (lavender).

In order to investigate the possible effects of the donor-only fraction on the fitting results, we built up artificial lifetime decays by adding random Poissonian noise to a generated model function (see Section 3.7). Therefore, models with two different mean inter-dye distances corresponding to a high FRET (40 Å) and a low FRET (60 Å) sample, a standard deviation of 6 Å , and a variable fraction of donor-only molecules were created. Subsequently, we fitted each decay to verify how different donor-only fractions and mean inter-dye distances affect both the accuracy and the precision of the estimated parameters. In order to simulate a real experiment, the starting guesses were set different from the true values. The results are reported in Figure 3B–D and Table A1 and Table A2 (see Appendix). In all of the analyzed decays, the estimated parameters are almost equal to the values obtained by starting the minimization from the true values. Therefore, the minimization algorithm (FMINUIT [24]) converged to the absolute minimum, independently of the fraction of donor-only molecules in the range from zero to 0.5. However, in the limit of error, the estimated parameters do not always correspond to the true values, as shown by the deviations from the yellow lines (see Figure 3B–D).

This observation can be explained by the strong anti-/correlation between the parameters. Indeed, one parameter may change with almost no effect on $\chi_{r}^{2}$ if there is a compensating variation of theanti-/correlated parameters. The anti-/correlation between the parameters of interest (*i.e.*, *x_D_*_0_, 〈*R_DA_*〉 and *σ_DA_*) is easily detected by visual inspection of the confidence surfaces calculated at one standard deviation (68.3%) from the minimum (see Section 3.6 for more details). A representative collection of these surfaces is reported in Figure 3A. Here, the characteristic banana-shape of the confidence surfaces indicates strong anti-/correlations between the parameters. The direction of the main axis, as well as the spatial extension of these surfaces change as a function of the true values in an unpredictable manner. However, a general trend is a relevant contraction of the confidence region, which is observed with decreasing donor-only fractions (compare the axes of the upper panels with the bottom panels in Figure 3A). This means that the parameters are recovered with higher precision at lower donor-only fractions, as convincingly shown in Figure 3E. Here, the relative errors (*i.e.*, the precisions) are reported for both the width *σ_DA_* and the mean 〈*R_DA_*〉 of the inter-dye distance distribution obtained from the high (pink and lavender) and the low (light blue and green) FRET efficiency-related lifetime decays. Overall, it is evident that the widths (light blue and pink) are recovered with lower precision with respect to the mean inter-dye distances shown in light green and lavender (with relative errors of *≤*40% compared to *≤*5%). Nevertheless, at high efficiency, the precision of *σ_DA_* is higher than at low efficiency (compare pink with light blue). Furthermore, the accuracy, *i.e.*, the deviation of the estimated parameter from the true value, can also be evaluated (see Figure 3F). The accuracy of the mean distance 〈*R_DA_*〉 is constant and always below 2% (see dashed lines). On the other hand, the accuracy of the width (continuous lines) strongly depends on the donor-only fraction and also on the mean distance. In more detail, for the high FRET states (pink), the accuracy is on average larger than for the low FRET states (light blue). In addition, dramatic drops in accuracy values, appearing at donor-only fractions between *∼*0.3 to *∼*0.1, are more pronounced for high FRET states (light blue). Therefore, depending on 〈*R_DA_*〉 and *x_D_*_0_, the accuracy may change in an unpredictable manner, which drastically complicates the data interpretation in real experiments where *x_D_*_0_ is generally unknown.

Altogether, these observations highlight that the donor-only fraction must be reduced as much as possible in order to increase the precision and, most importantly, the accuracy of the parameters of interest, *i.e.*, *σ_DA_* and 〈*R_DA_*〉. This requirement becomes impelling if the aim of the studies is to discriminate protein motions from linker dynamics. Indeed, a large deviation between the true and the estimated values is not tolerable if small contributions need to be identified and characterized. Thus, the donor-only fraction must be reduced below the highest limit of tolerance that we set at *∼*5%. In fact, this threshold value assures both the best precision (<15%) and accuracy (<10%) on the width *σ_DA_* and excellent precision (*<*1%) and accuracy (*<*0.5%) of the mean inter-dye distance 〈*R_DA_*〉, for both high and low FRET state-related lifetime decays. How to pursue this goal with single-molecule measurements is described in the following subsection.

### 2.2. Single-Molecule FRET-Filtered Lifetime Measurements

**Figure 4:**
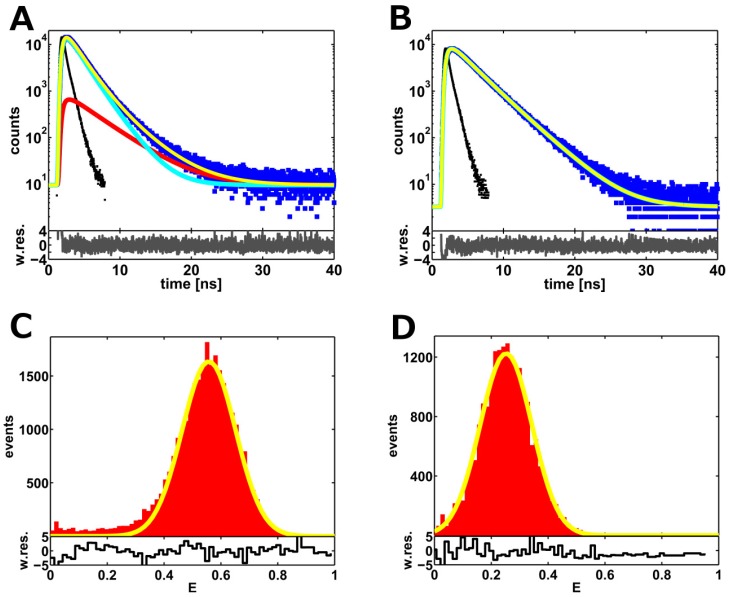
(**A**,**B**) FRET-filtered lifetime decays for 10-bp and 17-bp DNA samples. Here, the same color code is used as in Figure 3. The fitting parameters are reported in Table 1. (**C**,**D**) Efficiency histograms corresponding to the data given in the upper line are fitted with shot-noise limited Gaussian populations.

Here, we present a method to reduce the donor-only fraction below the limit of tolerance of *∼*5%, which is required to get precise and accurate inter-dye distance distributions from the fit of the donor lifetime decays (see Section 2.1). Single-molecule FRET (smFRET) measurements performed with pulsed interleaved excitation (PIE) on diffusing molecules are employed for this purpose. In fact, this excitation scheme permits one to filter out donor-only molecules from the collected single-molecule events [19]. In this way, lifetime decays without donor-only photons are obtained by adding up all of the selected donor photons. However, care must be taken here with respect to ordinary smFRET measurements in order to get a reasonable photon counting statistics, which should correspond to more than 10^4^ counts at the peak maximum of the TCSPC histogram. With the purpose of testing the method, we performed smFRET measurements with the DNA standards and compared the results with the ensemble experiments (see Figure 2). The fitting results are given in Table 1 and in Figure 4, where the corresponding transfer efficiency histograms are also reported. These results demonstrate that the filtering method is well suited to reduce the fraction of donor-only molecules below the limit of tolerance. For instance, the donor-only fractions decrease from *∼*17% in ensemble to *∼*5% (10-bp DNA sample) and to zero (17-bp DNA sample) for smFRET filtered data. The residual fraction of donor-only photons for the 10-bp DNA sample may be explained by multimolecule events and/or acceptor blinking, as shown by the typical low efficiency tail in the FRET histogram (see Figure 4C) [25,26]. Furthermore, both efficiency histograms are well fitted by shot-noise-limited Gaussian populations, as expected for fast inter-dye distance fluctuations [10].

The fact that the donor-only population is below the limit of tolerance for both samples ensures accurate values as obtained from the fits of smFRET filtered data. Thus, we can draw some general conclusions from the comparison with the ensemble experiments. First, we observe that the fitted mean inter-dye distances 〈*R_DA_*〉 slightly increase (by *∼*3 Å ), as compared to the values obtained previously from the ensemble experiments (see Table 1). The most probable explanation for this behavior is the correlation between the fitting parameters, as already mentioned above. On the other hand, for the widths, we obtain apparently inconsistent results. For instance, on the basis of the known three-dimensional structures of both labeled dsDNA samples (see Section 2.3), no difference is expected for the width parameters.  Nevertheless, the smFRET-filtered data reveal a lower value of (4.5 *±* 0.4) Å for 10-bp DNA as compared to the (7.2 *±* 0.5) Å obtained for the 17-bp DNA sample. A probable explanation for this observation is the base pair mismatch, which was introduced in proximity of the labeling position of the 10-bp DNA sample (see Section 3.1), although a satisfactory mechanistic explanation cannot be given at the moment. Indeed, two hypotheses can be proposed without really knowing which of them is most truthful. The mismatch can lead to a local distortion of the DNA structure, resulting in a structural rearrangement, which limits the donor accessible volume, either by (i) unspecific hard-sphere interactions and/or (ii) by more specific stacking interactions [27] between the dye and solvent-exposed DNA bases. The presence of a smaller second donor lifetime component for the 10-bp DNA sample as compared to the 17-bp DNA sample (see Table 2) would support the latter hypothesis. Indeed, it is known from experiments and MD simulations that fluorescence quenching may occur through photo-induced electron transfer (PET) mediated by ring-ring interactions [28]. On the opposite side, anisotropy measurements would support the first hypothesis. Indeed, the rotational freedom of the donor is apparently unperturbed with respect to the 17-bp DNA sample, as indicated by similar rotational correlation times at *∼*0.9 ns. The presence of local distortion in the 10-bp DNA structure is also reflected in the rather low mean efficiency value of *∼*0.55 (see Figure 4C), which is lower than the value of *∼*0.75 previously measured with a comparable DNA construct in our lab [17] and, independently, by Seidel and co-workers [9,29].

Remarkably, these observations demonstrate the higher sensitivity of single-molecule FRET filtered data, since the different widths between the 10-bp DNA and the 17-bp DNA distributions could not be resolved by the ensemble experiments, which gave rather similar values for both samples, *i.e.*, (7.1 *±* 0.3) Å and (5.9 *±* 0.5) Å. However, a good estimation of the distribution parameters was also obtained for the 17-bp DNA sample from the ensemble data (see Table 1).

### 2.3. A Weighted Accessible Volume Algorithm (wAV)

In this section, we first present the accessible volume (AV) algorithm [9], and subsequently, we show how to improve the model by introducing a proper weighting function of the calculated accessible points (wAV). This new algorithm is optimized for a precise estimation of the dye-linker amplitude of motions. Therefore, it is well suited to recover the amplitude of protein motions if combined with smFRET-filtered lifetime measurements. In order to test and calibrate the model, we used our DNA standards by comparing the measured inter-dye distance distribution with the one calculated with the wAV algorithm. Unfortunately, the 10-bp DNA sample has to be treated with reservation, because the effects of the base pair mismatch on the 3D structure and, consequently, on the wAVs are unknown.

**Figure 5:**
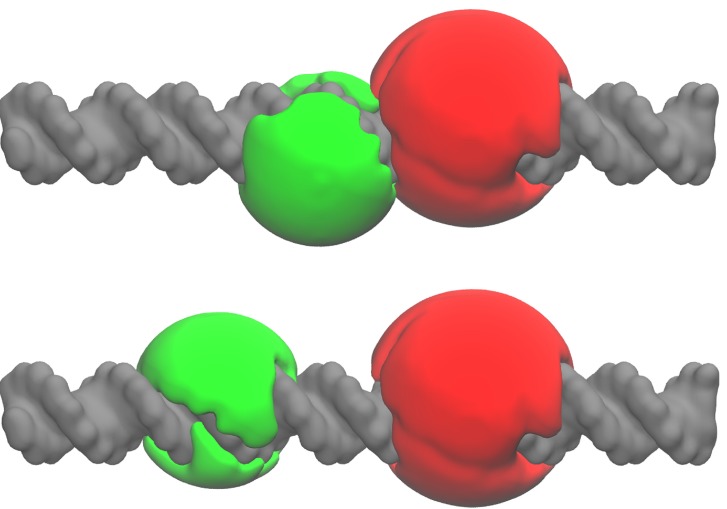
Surface representations of the calculated accessible volumes (AVs) for the 10-bp DNA (**top**) and the 17-bp DNA (**bottom**) structure. The AVs of the donor (T-C6-Alexa488) and the acceptor (T-C6-Alexa647) dye-linker constructs are displayed in green and in red, respectively. Although the size of the volumes can vary to a certain extend with parameters, such as the linker thickness, the results presented here are not significantly affected by them. A partial overlap between the AV clouds is observed for 10 bp DNA. The DNA structures were generated on the 3D-DART web server [30]. The representations were created with VMD (v.1.9).

Briefly, the AV algorithm calculates the sterically accessible positions determined by the spatial extension of the fluorescent probes and by the structure of the biomolecule (see Figure 5). Here, a coarse-grained description of the dye-linker construct is used where the dyes are represented as ellipsoids and the linkers as flexible tubes (for more details about the modeling, see Section 3.8). By using this algorithm, we assume that all points of the AV are equally accessible by the dye. Nevertheless, even if the AV algorithm can predict the mean distances 〈*R_DA_*〉 [9], the use of a homogeneous probability distribution within the AV is a rough approximation, leading to some problems concerning the width *σ_DA_* of the calculated inter-dye distance distributions. For example, for the 17-bp DNA sample, the calculated mean inter-dye distance of 62.6 Å is in good agreement with the experimental value of (62.7 *±* 0.2) Å, whilst the width (12.3 Å) is almost double in size as compared to the experimentally determined values of (7.2 *±* 0.5) Å. Additionally, the calculated distribution widths are sensitive to the grid spacing parameter employed for the calculation. For instance, a value of *∼*10.7 Å is obtained with a grid spacing of 0.4 Å [17]. Thus, a better-suited method is required to assess more realistic dye-linker contribution to the total amplitude of fluctuations obtained from the measurements. This can be achieved by modulating the dye accessibility to the AV points in order to consider hydrophobic and entropic effects. A Gaussian function centered at the attachment position turned out to be a reasonable and robust approximation of the real probability distribution (see Section 3.8). In addition, an ideal chain model [31] of the linker was used. These choices allow us to calibrate the wAV algorithm for a specificdye pair attached to DNA. Therefore, the effective bond length *beff* of the linker was varied untilthe widths $\sigma_{DA}^{wAV}$ of the calculated distance distributions were matching the values measured for the17-bp DNA sample. An additional check was performed by displacing the maximum of the probability distribution (given by Equation (13)) away from the labeled point. However, this approach overestimated *σ_AV_* for values of *b_ef__f_* larger than the thickness of the AV grid. Thus, this minimal set of parameters is enough for describing the effects of entropy and excluded volume, while additional interactions could be included in a later step, employing the more specific space-dependence of this probability distribution [20]. With the wAVs, an optimal effective length *b_eff _* = 3.6 Å and a mean distance 〈*R_DA_*〉= 61.2 Å , slightly smaller then the experimental value of (62.7 *± *0.2) Å , were obtained. On the opposite side, a calculated value of 40.6 Å, far smaller than the experimental determined value of (49.1 *± *0.5) Å, was obtained for 10-bp DNA, as well as a larger width of 6.9 Å with respect to the measured (4.5 *±* 0.5) Å. This can partially be explained by the aforementioned base pair mismatch, but an electrostatic repulsion between the negatively charged fluorophores (see Figure 1) may also contribute to increasing the measured distance with respect to the calculated one. In fact, as shown in Figure 5, the AVs partially overlap for 10 bp DNA.

Finally, once the distribution function has been parameterized by using a dsDNA calibration measurement, the obtained parameters can be utilized for application measurements with biological macromolecules under similar experimental conditions, assuming there is no sticking between the dyes and the molecule to which the dyes are attached. In this manner, the wAV can be used to disentangle the amplitude of the motions of flexible proteins from the total amplitude obtained from the experimental inter-dye distance distribution. This approach was already applied for studying the inter-domain motions of phosphoglycerate kinase (PGK) in combination with a coarse-grained simulation [17], neglecting the contribution of electrostatic interactions.

## 3. Experimental Section

### 3.1. Sample Preparation

Two double-labeled dsDNA samples were prepared, each single-strand labeled with either the donor (D) Alexa488 or the acceptor (A) Alexa647 dye. The distances between the dye labeling positions are 10 and 17 base pairs (bp). The dsDNA samples were assembled by hybridizing the single strands in annealing buffer (20 mM Tris, 100 mM NaCl, 10 mM MgCl2, pH 7.5) [9]. The individual strands were purchased from PURIMEX (Grebenstein, Germany) as follows: 5'-GGA CTA GTC TAG GCG AAC GTT TAA GG**X** GAT CTC TGT TTA CAA CTC CGA-3' (donor, 10 bp) and 5'-GGA CTA GTC TAG GCG AAC GTT TAA GGC GAT CTC **X**GT TTA CAA CTC CGA-3' (donor, 17 bp) with 5'-TCG GAG TTG TAA ACA GAG ATC **G**CC TTA AAC G**X**T CGC CTA GAC TAG TCC-3' (acceptor), where position **X** is a thymine bound at position 5' to the respective dye via a C6 amino linker. In the case of the 10-bp sample, a base pair mismatch was introduced in proximity of the labeled group at position 22 (**G**) of the donor strand in order to demonstrate the sensitivity of our method with respect to ensemble measurements. Single-labeled samples were put together by hybridizing the labeled strands with the complementary unlabeled strands (Eurofins MWG Operon, Ebersberg, Germany). For double-labeled samples, both strands were mixed together in a 1:1 ratio, whereas for single-labeled samples, the unlabeled strands were mixed in excess (1.2:1 ratio). Finally, the solutions were heated up to 98 *°*C and kept at this temperature for 3 min before they were cooled down to 25 *°*C with a gradient of $\frac{0.1{}^{\circ}C}{s}$ using a thermal cycler (PTC-200, MJ Research, USA). Successful hybridization was confirmed by gel electrophoresis.

### 3.2. Experimental Set-Up

Both, ensemble and single-molecule lifetime measurements were performed using a confocal microscope (Micro Time 200, PicoQuant, Berlin, Germany). Briefly, the light of two pulsed diode lasers operated at wavelengths of 481 nm and 633 nm (LDH-D-C 485 and LDH-D-C 640, PicoQuant, Berlin, Germany) is focused inside the sample solution by a water immersion objective (UplanSApo, 60*×*, NA 1.2, Olympus Deutschland, Hamburg, Germany). The emitted fluorescence light is separated from scattered excitation light by a dual-band dichroic mirror (XF2401, Omega Optical, Brattleboro, VT, USA), and focused on a 50 µm pinhole. Subsequently, the light is either separated by a dichroic mirror (620dcxr, Chroma Technology, Bellows Falls, VT, USA) into a donor and an acceptor detection channel, or split by a 50/50 beam splitter cube (U-MBF3, Olympus Deutschland, Hamburg, Germany). Finally, after passing the emission filters (FF01 530/55 for the donor and ET658/80M for the acceptor, Semrock Inc., Rochester, NY, USA), the light is detected by two silicon avalanche photodiodes (*𝜏*-SPAD, PicoQuant, Berlin, Germany). The lasers are operated at a repetition rate of 20 MHz using a computer-controlled laser driver (SepiaII, PicoQuant, Berlin, Germany), also enabling pulsed interleaved excitation [19] for single-molecule measurements (see Section 3.4). The data are processed by a time-correlated single photon counting (TCSPC) acquisition unit (PicoHarp-300, PicoQuant, Berlin, Germany) using a microtime resolution of 16 ps.

### 3.3. Ensemble Lifetime Measurements

Ensemble lifetime measurements were performed employing TCSPC. A data acquisition time was used, which ensured *∼*10^5^ counts at the peak maximum. The fluorescence detection rate was kept below 1% of the excitation rate (*i.e.*, 20 MHz) by employing fluorophores concentrations of a few nanomoles per liter in order to avoid the saturation of the detectors and pile-up effects [32]. The instrument response functions (IRFs) for excitation at 481 nm and 633 nm were measured by recording the emission of heavily-quenched dyes (Atto488-NHS and Atto655-NHS from ATTO-TEC, Siegen, Germany) in order to avoid color effects [33]. Therefore, the respective Atto dyes were diluted in a saturated potassium iodide solution [34] and recorded with a count rate of *∼*10% of the fluorescence detection rate of the actual sample until the maximum of the TCSPC histogram reached at least 10^4^ counts. The donor and acceptor lifetimes of single-labeled DNA were measured at ensemble level and used (i) for quantum yield determination [17] and (ii) as reference values for the FRET-quenched decays (see Section 3.5). These lifetime decays were recorded with two detectors using the 50/50 beam splitter. Since the difference between the lifetimes measured in the two channels was below *∼*1%, the resulting mean values were used for further analyses. On the opposite side, measurements of donor lifetimes in the presence of energy transfer were performed by employing a dichroic mirror. Indeed, even though a broader IRFwas observed, this allows one to compare ensemble experiments with single-molecule FRET-filtered measurements, where the same set-up was used (see Section 3.4). Full width half maximum values of *∼*448 ps and *∼*544 ps were obtained for the IRFs in both detection channels, respectively.

### 3.4. Single-Molecule Measurements

Single-molecule measurements were performed using a confocal microscope with pulsed interleaved excitation (PIE) [19] and separation of the donor emission from the acceptor emission by a dichroic mirror. The samples were diluted to an average number of *∼*0.03 molecules in the effective confocal volume. Single-molecule transits through the detection volume were identified with a threshold criterion applied to the inter-photon time-distance (IPD) trace [35,36] of the directly excited acceptor, also called the PIE channel. First, the IPD trace of the PIE channel was smoothed using a centered moving average filter with a width of 7 data points. This value has to be chosen carefully as a trade-off between suppression of background fluctuations [35] and smoothing down of smaller bursts. Secondly, an IPD threshold of 50 µs was used to detect fluorescence bursts corresponding to single-molecule detection events. Then, the beginning and ending of each burst were used to identify corresponding events in the donor and acceptor channels. This allows us to effectively filter out donor-only molecules and acceptor photobleaching events. Finally, only bursts with a total intensity *F_D_* + *F_A_* of at least 40 counts were retained for further analyses. The transfer efficiencies *E* were calculated burst-wise according to:

(1) $$E=\frac{F_{A}}{F_{A}+\gamma F_{D}}$$

where *F_D_* and *F_A_* are the burst-integrated counts of donor and acceptor photons and *γ* = *γʹ·g* = 0.47 is a correction factor accounting for quantum yield differences between the two dyes and detection efficiency mismatches between the detection channels. The quantum yield ratio *γʹ* = $\frac{\phi A}{\phi D}$ = 0.36 was determinedby lifetime measurements of the single-labeled samples (see Table 2).

**Table 2:** Summary of the measured fluorescence lifetimes and quantum yields of single-labeled dsDNA samples (D0, donor only; A0, acceptor only) and free dyes. The quantum yields of the free dyes are the ones given by the producers. The lifetime decays were fitted with a multi-exponential model function (see Equation (6)). The amplitude *x_i_* and lifetime *𝜏_i_* of each component are given, as well as the reduced *χ*^2^ values.

Sample	*𝜏*_1_	*𝜏*_2_	*x*_1_	$\chi_{r}^{2}$	*ɸ*
D0-dsDNA (10 bp)	4.06 ns	1.38 ns	0.94	1.07	0.90
D0-dsDNA (17 bp)	4.09 ns	1.52 ns	0.91	1.04	0.88
A0-dsDNA (10 bp and 17 bp)	1.38 ns	0.88 ns	0.28	1.10	0.32
Alexa 488-NHS	4.025 ns	-	1	1.13	0.92
Alexa 647-NHS	1.053 ns	-	1	1.80	0.33

Finally, the detection efficiency ratio *g* = $\frac{g_{A}}{g_{D}}$ = 1.3 was obtained as described in [37]. The measuredburst-integrated fluorescence intensities *FD* and *FA* were determined by subtracting background counts *BG_D_* and *BG_A_* from the measured intensities *S_D_* and *S_A_* and by correcting for donor cross-talk (*α* = 0.85%):

(2) $$F_{D}=S_{D}-BG_{D}$$

(3) $$F_{A}=S_{A}-BG_{A}-\alpha \cdot F_{D}$$

### 3.5. Data Analysis

Data analysis was performed with self-written MATLAB (v.R2011a, 64 bit) routines. Fits were performed by least-squares minimization [38]. Minimization was carried out with the MINIMIZE command of the FMINUIT package bound to MATLAB [24]. The minimization was run until the estimated vertical distance from the minimum was less than 10^−8^. Values of the reduced *χ*^2^ [38] were employed to assess the goodness of fit. Calculated efficiencies obtained from individual bursts were used to build FRET histograms, which were subsequently fitted with Gaussian distribution functions parameterized by the mean 〈*E*〉 and the variance $\sigma_{E}^{2}$. Shot-noise limited populations were evaluated by setting the variance to the values calculated with the following expression [10,26]:

(4) $$\sigma_{SN}^{2}=\frac{\langle E\rangle \cdot (1-\langle E\rangle )}{N_{T}}$$

Here, the threshold *N_T_* applied to the total number of photons in one burst was used for the calculation Of $\sigma_{SN}^{2}$ instead of the more common average number of photons 〈*N*〉. This choice is justified by the fact that 〈*N*〉 (*∼* 75) is not too far from *N_T_* (40). In a second evaluation, lifetime decays without donor-only photons were built by binning all of the donor microtimes selected by the analysis of the single-molecule FRET experiments. Here, the corresponding IRF was measured at a reduced count rate. The measured lifetime decays *I*(*t*) were fitted by performing an iterative reconvolution of the measured *IRF*(*t*) and the chosen model function *F* (*t*) [39]:

(5) I(t)=IRF(t)∗F(t)

For single-labeled DNA and free dyes, a multi-exponential model function was used to account for local quenching:

(6) $$F(t)=I_{0}\cdot \sum_{i}x_{i}\cdot e^{-\frac{t}{\tau_{i}}}$$

where *I*_0_ is the maximum intensity, *x_i_* is the amplitude fraction and *𝜏_i_* is the lifetime of the *i* *–* *th* component. The results for free dyes and for single-labeled DNA are given in Table 2. The quantum yields of single-labeled DNA were calculated from the amplitude averaged lifetimes [17] using the quantum yields of the free dyes (Alexa 488-NHS and Alexa 647-NHS) as references. In the presence of the acceptor dye, each donor lifetime component (see Equation (6)) is quenched by FRET as a function of the inter-dye distance *R_DA_* [37]. Therefore, the total decay *F_DA_*(*t*) is the sum of distance-dependent multi-exponential lifetime decays weighted by the probability *p*(*R_DA_*) to find the dyes at distance *R_DA_*:

(7) $$F_{DA}(t)=\sum_{i}x_{i}\int_{R_{DA}}p(R_{DA})\cdot e^{-\frac{t}{\tau_{D0,i}}\left[1+{\left(\frac{R_{0}}{R_{DA}}\right)}^{6}\right]}dR_{DA}$$

where the Förster radii ($R_{0}^{10bp}$ = 53.1 Å and $R_{0}^{17bp}$ = 53.6 Å) were calculated according to [1] by using $\epsilon_{A}^{\max}$ = 270,000 *M*^–1^ *cm*^–1^ and *ϕ*_D_ = 0.92. In this model, the physical information about dye motions is enclosed within the probability distribution *p*(*R_DA_*), which depends on the portion of space accessible to the dyes and on their relative configuration. In general, it turns out that this distribution is well approximated by a Gaussian function [9]:

(8) $$p(R_{DA})=\frac{1}{\sqrt{2\pi }\cdot \sigma_{DA}}\cdot e^{-\frac{{\left(R_{DA}-\langle R_{DA}\rangle \right)}^{2}}{2\cdot \sigma_{DA}^{2}}}$$

where the standard deviation *σ_DA_* describes the average amplitude of the inter-dye distance fluctuations and the mean 〈*R_DA_*〉 the most probable inter-dye distance. Therefore, the total model function for the donor fluorescence intensity in the presence of the acceptor becomes:

(9) $$F(t)=I_{0}\left[\left(1-x_{D0})F_{DA}(t)+x_{D0}F_{D0}(t\right)\right]+BG$$

here, a donor-only fraction *x_D_*_0_ and a constant background *BG* are also considered. Possible origins of the donor only fraction are inactive and missing acceptors and/or acceptor blinking. This model function was employed to fit the experimental donor lifetime decays by numerically integrating Equation (7) in an interval of [〈*R_DA_*〉*−* 4*σ_D__A_...* 〈*R_DA_*〉 + 4*σ_D__A_*] with a step size of 0.25 Å. All of these fits were performed in a time interval of the TCSPC histogram starting approximately 1 ns before the peak maximum position and ending at 40 ns. The corresponding results are reported in Table 1 for ensemble and single-molecule filtered data.

### 3.6. Error Analysis

The confidence surfaces in the parameter space were determined as follow. The analysis was restricted to the three parameters of physical interest {*ξ*}= {*σ_DA_,*〈*R_DA_*〉*,x_D_*_0_}. First, the function $\chi_{r}^{2}$(*ξ*) was calculated on a sufficiently dense three-dimensional grid of points centered around the minimum coordinates obtained from the fit. The remaining parameters (*i.e.*, *I*_0_ and *BG*) were fixed. Second, the ratio $\chi_{r}^{2}(\xi )/\chi_{r,\min}^{2}$ between the function $\chi_{r}^{2}(\xi )$ and the value $\chi_{r,\min}^{2}$ assumed by the function at the minimum was calculated. Then, the F-distribution was employed to determine the confidence surface [1,40]:

(10) $$\frac{\chi_{r}^{2}(\xi )}{\chi_{r,\min}^{2}}=F_{\chi }\left(k,v,p\right)$$

where *k* is the number of parameters, *ν* gives the degrees of freedom and *P* is the probability that the value of *F_χ_* is due to random fluctuations. In order to have a confidence level of 68.3% corresponding to one standard deviation error, *P* was set to 0.32. The confidence intervals on each estimated parameter were obtained with a support plane analysis [40]. Here, one parameter was systematically changed over an interval centered around the estimated value. For each value of this interval, all other parameter where allowed to change in a minimization run, and the minimum $\chi_{r}^{2}$ were stored. The confidence intervals are obtained by plotting the $\chi_{r}^{2}$ as a function of the fixed parameter values.

### 3.7. Generation of Artificial Lifetime Decays

Artificial lifetime decays were generated by the use of MATLAB routines (v.R2014a, 64 bit). For this purpose, random Poissonian noise was added to a set of model functions generated with Equation (9). The decays were employed to determine the accuracy and the precision of the estimated parameters in the presence of a variable fraction of donor-only molecules and as a function of the mean inter-dye distance. Therefore, inter-dye distance distributions were used having a mean 〈*R_DA_*〉 = 40*/*60 Å (high/low FRET) and a width *σ_DA_* = 6 Å comparable to those experimentally determined from the high/low efficiency DNA samples. Variable fractions *x_D_*_0_ (0%, 5%, 10%, 20%, 30%, 40%, 50%) of donor-only molecules were also added to the FRET quenched lifetime decays. The set of artificial lifetime decays was fitted with the same model function employed for data generation. Here, in order to simulate a real experiment, where the true values are unknown, the start guesses were set to (i) 〈*R_DA_*〉 = 35*/*53 Å, (ii) *σ_DA_* = 8 Å and (iii) 0.9 *·* *x_D_*_0_. However, since the amplitude at time zero *I*_0_ and the constant background *BG* are easily retrieved from the experiments, these values were set to the true values, which are *I*_0_ = 10^4^ and *BG* = 20. The goodness of fit was evaluated from the $\chi_{r}^{2}$ distribution. As an outcome of these fits, the following mean $\chi_{r}^{2}$ values were obtained: $\langle \chi_{r}^{2}\rangle$ = 1.033 (for 60 Å) and $\langle \chi_{r}^{2}\rangle$ = 1.056 (for 40 Å). The resulting distribution parameters are reported in Figure 3, whereas the whole set of fitting parameters is given in Table A1 and Table A2 (see the Appendix). To analyze whether the minimization algorithm converged to the absolute minimum, the fits were repeated by using the true values as starting guesses. No difference was observed in the estimated parameters, indicating a correct convergence criterion (see Section 3.5). The precision of the estimated parameters was evaluated with the relative errors *δξ/ξ*. The accuracy was calculated as the relative difference (*ξ − ξ_true_*)*/ξ_true_* between the estimated and the true values.

### 3.8. Weighted Accessible Volume Algorithm

Initially, the accessible volume (AV) algorithm [9] generates for each fluorophore a cloud of sterically accessible points around the labeled group from a given macromolecule configuration. Therefore, the 3D structures of both dsDNA samples were generated with the 3D-DART (3DNA-Driven DNA Analysis and Rebuilding Tool) web server [30].  Afterwards, the mean distance 〈*R_DA_*〉 between the dyes was obtained by averaging over all accessible points of both donor and acceptor:

(11) $$\langle R_{DA}\rangle =\frac{1}{MN}\cdot \sum_{i}^{M}\sum_{j}^{N}\left|R_{D,i}-R_{A,j}\right|$$

here, **R_D_** and **R_A_** are the individual donor and acceptor accessible positions. In addition, the width of the inter-dye distance distribution was calculated using the definition of the standard deviation:

(12) $$\sigma_{DA}=\sqrt{\langle R_{DA}^{2}\rangle -{\langle R_{DA}\rangle }^{2}}$$

Apparently, *σ_DA_* is overestimated by Equation (12), because all points inside the AV are considered to be equally occupied. The model can be improved by weighting each AV point **R** by a semi-empirical Gaussian distribution:

(13) $$p(R)={\left(\int_{AV}e^{-\frac{{\left(R-R_{\text{attach}}\right)}^{2}}{2\sigma_{AV}^{2}}}dR\right)}^{-1}\cdot e^{-\frac{{\left(R-R_{\text{attach}}\right)}^{2}}{2\sigma_{AV}^{2}}}$$

where **R_attach_** is the attachment position of the linker to the  macromolecule.  Accordingly, Equations (11) and (12) were recalculated using the weighted accessible volumes (wAV), which penalize the fully-extended conformation of the linker due to the hydrophobicity [20] and conformational entropy [9] of the aliphatic chain. The width *σ_AV_* of the AV weighting function was derived from a Gaussian chain model [31] of the linker, which yields an average end-to-end distance of:

(14) $$\sigma_{AV}=\sqrt{\frac{b_{eff}\cdot L_{link}}{3}}$$

where *b_eff_* is an effective bond length and *L_link_* is the contour length of the linker. The latter is taken as the distance between the labeled atom and the center of the dye with the linker in an extended conformation (see Figure 1). However, other variations of these dependences are possible. For instance, if the linker is taken as rigid, the width *σ_AV_* scales with the contour length *L_link_* instead, like $\sqrt{L_{link}}$. The difference of calculated *σ_AV_* values obtained for both scenarios is *∼*20% in the worst case, as shown for phosphoglycerate kinase [17]. Therefore, once the effective bond length is calibrated for a specific dye pair by using an intrinsically rigid standard (dsDNA in our case, but polyproline helices can in principle also be used), this choice allows one to scale the width *σ_AV_* of the weighting function to account for different effective bond lengths.

In our work, a C-based code was written to compute the wAVs. In order to do so, the AVs were calculated first by coarse graining the dyes as ellipsoids with semiaxes *R_dye,_*_1_, *R_dye,_*_2_ and *R_dye,_*_3_ and the linkers as flexible tubes, described by the width *w_link_* and the length *L_link_* (see Table 3). These geometrical parameters were derived from the chemical structures generated by ChemDraw (v.14), which are shown in Figure 1. The grid spacing employed for the calculation was 0.8 Å, a larger value with respect to [17], where a grid spacing of 0.4 Å was used. This was done to speed up the calculations. Effectively, a coarser grid increased the distribution width *σ_DA_* by *∼* 12% without substantially changing the mean 〈*R_DA_*〉. The wAVs were calculated by systematically varying the effective bond length *beff*until the experimentally-determined *σ_DA_* matches the calculated value of $\sigma_{DA}^{wAV}$ (see Section 2.3 for more details).

**Table 3:** Dimensions of the dye-linker constructs for dyes attached to DNA at position 5' of a thymine (T) via a C6 amino linker. Notation according to 15.

Dye	*L_link_*	*w_link_*	*R_dye,__1_*	*R_dye,__2_* **	*R* *_dye,_* *_3_*
T-C6-Alexa 488	19.3 Å	4.5 Å	5.2 Å	4.2 Å	1.5 Å
				
T-C6-Alexa 647	24.6 Å	4.5 Å	9.9 Å	7.7 Å	1.5 Å

## 4. Conclusions

The inter-dye distance distribution obtained from lifetime FRET measurements is a precious source of information about the static and dynamic properties of biomolecules. For example, details about average conformations and amplitudes of fluctuations at the equilibrium can in principle be obtained from these measurements. However, the information is often entangled with the dye-linker motions, in particular if fast protein dynamics occurs (*∼*100 ns). In addition, ordinary ensemble lifetime data typically suffer from the presence of donor-only molecules, precluding an accurate and precise estimation of the inter-dye distance distribution parameters. In the present work, we demonstrate that recovering inter-dye distance distribution from smFRET-filtered lifetime decays is a powerful approach to overcome the above-mentioned limitations. Indeed, the use of pulsed interleaved excitation allowed us to reduce the fraction of donor-only molecules below the determined limit of tolerance of *∼*5%, which is required to extract reliable fitting parameters from the data. Moreover, the filtering method can in principle be extended to the analysis of any sub-ensemble of molecules fulfilling a predetermined selection criterion based on the measured physical properties (efficiency, lifetime, anisotropy, burst duration, *etc.*). This allows one also to filter out donor-only molecules, even in the absence of pulsed interleaved excitation. Recently, alternative approaches based on ensemble lifetime FRET measurements were used to characterize the distance distribution for two conformational states in a human guanylate binding protein in the presence of large donor-only fractions of about 70% [41]. In that work, the parameters were determined by sophisticated minimization routines. Namely, a Markov chain Monte Carlo sampling was performed by using the Metropolis–Hastings algorithm. Nevertheless, we think that our approach may facilitate data fitting by ruling out correlation effects between the parameters and data overfitting. For instance, in the aforementioned work, a global width was employed for two Gaussian inter-dye distance distributions; whereas, with smFRET filtered lifetime decays, it may be possible to recover the width of each Gaussian independently. Additionally, with our method, it is in principle feasible to combine the information from the efficiency histograms and the one from the filtered lifetime decays by means of global fits. In this respect, different analysis schemes can be envisioned. For instance, either the occupancy of each population or the mean distances or even both can be used as global parameters. In this way, the species sensitivity of smFRET measurements would be combined with the high spatial and temporal resolution of lifetime measurements.

For completeness, we should also mention the limitations of the proposed approach. First, the necessity to acquire a large number of donor photons (*∼*10^6^) at low count rates of *∼*2 KHz typical of single molecule measurements leads to long measuring times, which may cause trouble with unstable samples. Secondly, the broader IRFs of single-photon avalanche photodiodes (SPAD) as compared to the photomultiplier detectors (PMT), the latter often used in ensemble measurements, may introduce larger uncertainties on the estimated parameters at the short lifetimes that correspond to high FRET states. However, this effect is probably compensated for by the higher precision and accuracy of the estimated parameters reached with high FRET states with respect to low FRET populations (see Figure 3E,F). Moreover, further developments can be envisioned to improve the proposed coarse-grained model. For instance, electrostatic interaction may play a role at short distances where the dyes can mutually repel/attract each other. In addition, electrostatic interactions of the dye with the biomoleculemay distort the probability distribution, especially for proteins. Therefore, it may be necessary to improve the wAV model by considering different weighting functions accounting for the electrostatic forces. However, the wAVs resemble the real physical distribution well enough to allow a robust interpretation of the data in the case of PGK [17] and DNA.

To conclude, we think that the proposed combination of smFRET-filtered lifetime measurements and a wAV algorithm is a promising tool. In this regard, the method is perfectly suited to study the amplitudes of the motions of biomolecules, in general, and, more specifically, fast fluctuation amplitudes in combination with other techniques. For instance, conformational fluctuations on characteristic timescales of nanoseconds can be obtained from time-resolved correlation analyses [42], while computer simulations give mechanistic insights about these motions.

## Appendix

**Table A1:** Fitting parameters, as obtained from the fits of the artificial time decays, as well as the true values (first column and last line) used to build the model functions. The confidence intervals at one standard deviation (68.3%) where calculated with support plane analysis. In more detail, *I*_0_ is the amplitude at time zero, *B**G* is the constant background, *x**_D_*_0_ is the donor-only fraction, 〈*R_DA_*〉is the mean inter-dye distance and *σ**_DA_* is the width of the inter-dye distance distribution. Here, a high FRET sample with a true value of the mean inter-dye distance of 〈*R_DA_*〉 = 40 Å is used. For more information, see Section 3.6 and Section 3.7.

$x_{D0}^{true}$(%)	*I*_0_ (counts)	*BG* (*counts*)	*x_D_*_0_ (%)	〈 *R_DA_*〉 (Å)	$\sigma_{DA}$(Å)	$\chi_{r}^{2}$
50	9*,***999	18.8	49 (46 *–*** *–* 52)	39.5 (37.8 *– –* 40.7)	6.2 (4.8 *– –* 7.5)	1.097
						
40	10*,***005	18.8	40 (38 *– –* 42)	40.1 (39.0 *– –* 40.1)	5.8 (4.8 *– –* 7.5)	1.080
30	9*,***998	19.3	32 (31 *– –* 33)	40.8 (40.3 *– –* 41.2)	5.4 (4.7 *– –* 5.9)	1.032
20	9*,***986	18.8	21 (20 *– –* 21)	40.5 (40.2 *– –* 40.1)	5.7 (5.3 *– –* 6.1)	1.049
10	9*,***974	18.9	10 (9.6 *–* ** *–* 10.3)	40.0 (39.7 *– –* 40.3)	6.0 (5.6 *– –* 6.3)	1.019
5	9*,***986	18.8	5 (4.9 *– –* 5.2)	39.9 (39.7 *– –* 40.2)	6.0 (5.8 *– –* 6.3)	1.0092
0	10*,***028	19.0	0 (0.0 *– –* 0.0)	40.1 (39.8 *– –* 40.3)	5.9 (5.8 *– –* 6.1)	1.0025
true values	10*,*000	20	*/*	40.0	6.0	*/*

**Table A2:** Same fitting parameters as described in Table A1. The true value of the mean inter-dye distance is 〈*R_DA_*〉 = 60 Å which corresponds to a low FRET sample.

$x_{D0}^{true}$(%)	*I*_0_ (counts)	*BG* (*counts*)	*x_D_*_0_ (%)	〈 *R_DA_*〉 (Å)	$\sigma_{DA}$(Å)	$\chi_{r}^{2}$
50	9*,*997	19.1	51 (47 *– –* 54)	59.9 (58.7 *– –* 61.3)	5.3 (3.0 *– –* 7.3)	1.019
40	10*,*001	18.8	41 (38 *– –* 44)	59.5 (58.6 *– –* 60.6)	5.4 (3.8 *– –* 7.0)	1.050
30	10*,*004	19.0	31 (28 *– –* 34)	59.8 (59.1 *– –* 60.7)	5.6 (4.4 *– –* 6.8)	1.008
20	10*,***011	18.8	22 (19 *– –* 25)	59.5 (58.9 *– –* 60.3)	5.4 (4.2 *– –* 6.5)	1.037
10	9*,***986	18.6	14 (11 *– –* 16)	59.3 (58.8 *– –* 59.9)	4.6 (3.7 *– –* 5.5)	1.041
5	9*,***992	18.7	7 (4 .6 *–* ** *–* 9.0)	59.7 (59.2 *– –* 60.3)	5.4 (4.4 *– –* 6.2)	1.037
0	9*,***992	18.8	0 (*–* 0.3 *– –* 0.2)	60.0 (59.6 *– –* 60.6)	5.9 (5.2 *– –* 6.7)	1.041
true values	10*,*000	20	*/*	60.0	6.0	*/*
